# Label-Free Fried Starchy Matrix: Investigation by Harmonic Generation Microscopy

**DOI:** 10.3390/s19092024

**Published:** 2019-04-30

**Authors:** Agathe Chouët, Sylvie Chevallier, Romain Fleurisson, Catherine Loisel, Laurence Dubreil

**Affiliations:** 1 Oniris, Univ Nantes, CNRS, GEPEA, UMR 6144, F-44000 Nantes, France; agathe.chouet@oniris-nantes.fr (A.C.); catherine.loisel@oniris-nantes.fr (C.L.); 2 PAnTher, Oniris, INRA, Université Bretagne Loire, F-44307 Nantes, France; romain.fleurisson@oniris-nantes.fr

**Keywords:** multiphoton microscopy, starch granule, topography, harmonic generation, SHG, THG

## Abstract

An innovative methodology based on non-destructive observation by using harmonic generation microscopy is proposed for detection and location of starch granules and oil in a fried starchy matrix and topography analysis of food products. Specific fluorescent probes were used to label the main biochemical components of the starchy fried matrix, namely starch and oil. Fluorescence of starch and oil respectively stained with Safranin O and Nile red was observed from non-linear microscopy. By using sequential scanning and specific emission filters, it was possible to merge fluorescence and harmonic generation signals. Second harmonic generation (SHG) generated by starch granules was superposed with safranin fluorescence, whereas third harmonic generation (THG), not restricted to the superposition with Nile red fluorescent signal, was used to investigate the topography of the fried product. By these experiments, starch granule mapping and topography of the starchy fried product were obtained without any destructive preparation of the sample. This label-free approach using harmonic generation microscopy is a very promising methodology for microstructure investigation of a large panel of starchy food products.

## 1. Introduction

The distribution of constituents in the matrix of fried foods and the topography of the product are important information to understand phenomena occurring during the frying process. Penetration of oil in the matrix and distribution of non-gelatinized starch are two phenomena usually analyzed in fried products. Bouchon and Pyle [1] highlighted a relationship between those two components and their importance on the quality of fried products. They demonstrated that presence of native starch in potato snacks could cause the increase of oil in the product, and the decrease of the surface roughness. 

According to Pedreschi [2], oil absorbed during the cooling step is the major part of the total oil content in potato chips. However, oil uptake is not only due to the absorption during the cooling step. Bouchon et al. [3] showed that three different absorption mechanisms exist for fried potato cylinder that lead to three different oil fractions. These three fractions are (i) oil absorbed during frying, (ii) oil absorbed during cooling by a pressure gradient created by vapor condensation and surface tension forces, and (iii) oil on the surface favored by product roughness [1]. Oil absorption is essentially a surface phenomenon: Rubnov and Saguy [4] demonstrated the relationship between this phenomenon and the surface roughness of the fried restructured potato products, and Rahimi and Ngadi [5] demonstrated the positive correlation between the roughness and the surface fat content of fried batters. These studies showed that oil on the surface of the product, absorbed during the cooling step is related to topographic attributes of the product.

However, Thanatuksorn et al. [6] did not find any correlation between surface roughness and oil uptake in wheat flour-based products. Moreno et al. [7] reported that the roughness of the product is an important factor in oil absorption, but other properties of the products are involved, such as the formulation or the product type (potato or gluten-based product).

Development of surface structure and roughness of fried products is mostly due to the crust formation that creates an increase of the pressure inside the product. This pressure leads to expansion of pores and could generate damage to the surface of the product [5].

Topography analysis could be performed by using scanning electron microscopy (SEM). Lisinska and Golubowska [8] used images obtained by SEM to compare surfaces of French fries at different steps of the production. Rahimi and Ngadi [5] and Rubnov and Saguy [4] analyzed the surface roughness of the product by measuring the fractal dimension from images obtained by SEM. Fractal dimension is measured as the degree of irregularity and its value is interpreted as a surface roughness index. Bouchon and Pyle [1] characterized the surface of restructured potato chips by using reflective confocal laser scanning microscopy (CLSM). They obtained a color-coded map in gray scale with different gray levels for each height. The surface roughness descriptors were determined from the height elevation matrix. Moreno et al. [7] used the scanning laser microscopy (SLM) to analyze product topography. This method allowed analyzing the surface without contact between instrument and product. SLM is a profiling instrument that measures a set of heights as functions of position that can be used to construct a topographic map.

Oil impregnation is usually analyzed by a microscopic observation. In the majority of studies, a dyed oil bath with a thermostable fluorescent probe is used in order to label absorbed oil in the product during the frying process [2,9,10,11]. In addition to fluorescent labeling with Nile red, Pedreschi and Aguilera [9] used CLSM in fluorescence mode to observe the product and reconstruct in 3D an image of oil distribution in potato chips. Vauvre et al. [10] used micro-tomography or CLSM coupled to dynamic observation of oil by illumination of the sample with a synchrotron source at 295 nm. Signals from fluorescence of oils were isolated by segmentation or image subtraction. 

Starch gelatinization evaluation is usually made by light microscopy or differential scanning calorimetry (DSC) for observation or quantification of the starch gelatinization respectively [12]. Light microscopy with polarization allows observation of the distribution of native wheat starch granules in the matrix due to their birefringence. Light microscopy requires a preparation of samples to obtain a thin section of the product. In a fried starchy matrix, additional treatments are usually necessary such as inclusion in a specific medium in order to achieve thin sections of the product. All these treatments may induce a noticeable modification of the structure or of the distribution of oil and starch granules in the matrix [11,12]. In contrast to light microscopy, DSC only allows quantifying the gelatinization level by determining the enthalpy of gelatinization reactions corresponding to the starch in the matrix that will gelatinize in excess of water. This method does not give information about starch repartition in the matrix, but can give information about the level of gelatinization of starch in the product before and after the frying process. In addition, scanning electron microscopy is commonly used to observe starch on the surface of the analyzed product [8,13,14,15,16]. In order to have better observation of the fried product, this method requires a preliminary treatment of defatting in petroleum or acetone [13,17] or a lyophilization step [16,18]. SEM is restricted to observation of native starch granules to the surface of the product. In 2010, by the observation of fresh potato tissue, Achir et al. [19] showed that it was possible to observe potato starch granules in the cell by CLSM in fluorescence mode without specific staining. It was assumed that the cell walls of potato had phenolic compounds that could emit auto fluorescence. 

Recently, second harmonic generation (SHG) and third harmonic generation (THG) obtained from nonlinear microscopy have been described as specific signals to identify label-free components in many biological samples [20,21,22,23,24,25]. SHG results from the conversion of two incoming photons into one emitted photon with doubled energy and occurs at ordered non-centrosymmetric structures that are found predominantly in crystals [26]. Then, Mizutani et al. [23] demonstrated that starch granules could be detected in living plants based on their SHG signals. Débarre et al. [21] also showed the interest of SHG to detect starch granules in plant seeds. Zhou et al. [27] demonstrated that SHG was mainly generated by amylopectin. Cox [21] used SHG to detect starch granules in starchy food because of the strong SHG signal given by starch in its crystalline form, and highlighted the interest of this technique in food industry to follow recrystallization of starch in order to prevent defects or improve texture of products. The hyperpolarizability of individual molecules of glucose and amylopectin was calculated by Cisek et al. [24] and found to be extremely small. It was concluded that the anisotropic water molecules trapped in the crystals were responsible for SHG in starch, explaining the hydration effect observed [25,26,28]. 

THG was also explored for mapping optical heterogeneities in biological samples [29,30]. THG occurs at structural interfaces, such as local transitions of the refractive index whereby the combined energy of three photons is converted into one emitted photon with one third of the excitation wavelength and tripled energy [31]. Débarre et al. [21] showed the possibility to image the distribution of lipids in liver tissue and lipid rich bodies in plant seeds from THG. In another biological context, Dubreil et al. [22] demonstrated the interest of the THG detection from micrometer size lipid in adipocytes and dystrophic muscle. Recent publications have also shown that THG imaging could be powerful technique to investigate microstructure based on the refractive index heterogeneities in cells and tissues [32,33].

The objective of our study was to introduce nonlinear microscopy as a new methodology for structure analysis of a label-free potato fried starchy matrix by investigating the auto fluorescence, SHG from starch granule and THG from interfaces heterogeneities (lipid and surface).

## 2. Materials and Methods

### 2.1. Sample Preparation

The matrix used in this study was prepared from a mix of dehydrated potato and native starch. Dough was formed by mixing these dry ingredients with water. Dough was sheeted using a rolling mill and pieces were cut in the sheeted dough. Pieces of dough were fried at 170 °C in a bath of sunflower oil.

Specific staining of starch was performed to validate the harmonic signals attribution to starch granules (SHG). Safranin-O (Sigma Chemical Co.) was prepared at 1 mg/mL in water to stain the starch granules. Safranin-O is not specific to starch but it does not show fluorescence in the absence of starch, so, it has become a starch marker [34]. We added 0.1 mL of solution of Safranin-O to (i) native starch preparation, (ii) the fried starchy product. Safranin-O emission wavelengths are reported in Table 1.

Oil staining was performed by using Nile red (Sigma Chemical Co.) at 1mg/mL in acetone to stain (i) the lipid droplets obtained from sunflower oil/water emulsion (continuous phase water) (ii) the lipid phase of the fried product. We added 0.05 mL of Nile red solution on pieces of 0.5 cm × 0.5 cm of starchy fried matrix. Fluorescence emission wavelength of Nile red is reported in Table 1. Fried starchy samples were immobilized between glass slide and cover glass separated by spacers to avoid the crushing of it. No mounting medium was used to preserve the structure of the fried dry sample.

### 2.2. Multiphoton Microscopy

Measurements were performed on a Nikon microscope A1RMP-HD coupled with an Insight Deepsee laser (Spectra Physics), used in the 820−1300 nm range <120 fs pulse width (APEX platform of the INRA/Oniris UMR 703 PAnTher Nantes, France, Center of Excellence Nikon Nantes). An auxiliary beam at 1040 nm was available in combination with the tunable output for dual wavelength excitation. For all images, an apochromat 25X MP1300 immersion objective was employed (NA 1.10, WD 2.0 mm) and distilled water was used as the immersion medium. The microscope was equipped with four non-descanned detectors installed in reflexion mode, three of them were highly sensitive GaAsPs (blue, green, and yellow channels). THG and SHG were performed in epi directions. Different dichroic and emission filters from Semrock were used according to excitation wavelengths and analyzed signals (fluorescence and harmonic signals) (Table 2).

**Figure 1:**
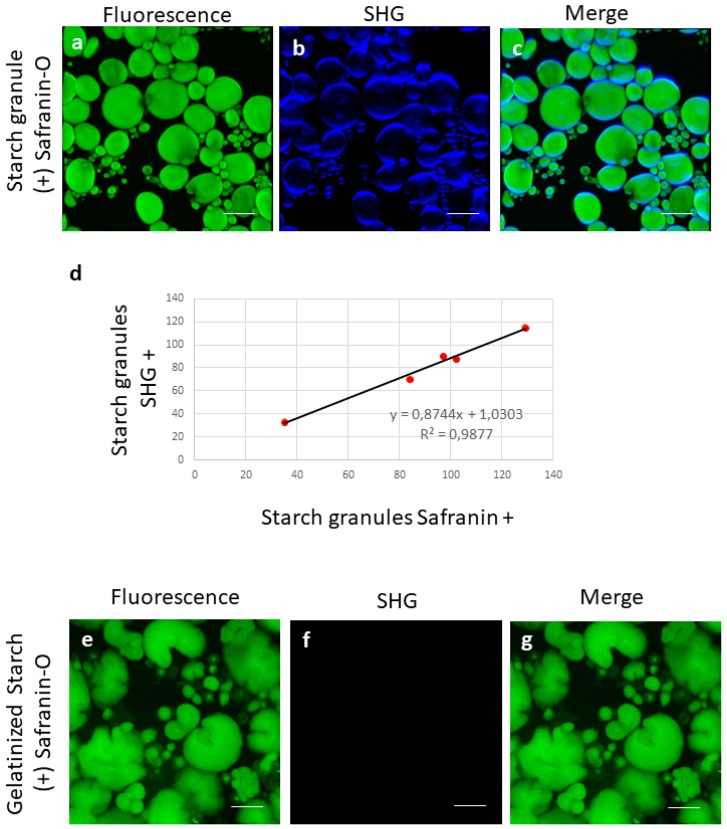
(**a**–**c**) Observation of native starch granule stained with Safranin-O: (**a**) Safranin-O fluorescence signal (green); (**b**) second harmonic generation (SHG) signal (blue); (**c**) superposition of SHG and fluorescence signals; (**d**) comparison SHG/fluo starch granule detection; (**e**–**g**) Observation of gelatinized starch granule stained with Safranin-O: (**e**) Safranin-O fluorescence signal (green); (**f**) absence of SHG in gelatinized starch; (**g**) superposition of (**d**) and (**e**). Scale bar 25 µm. See Table 2 for acquisition parameters.

**Figure 2:**
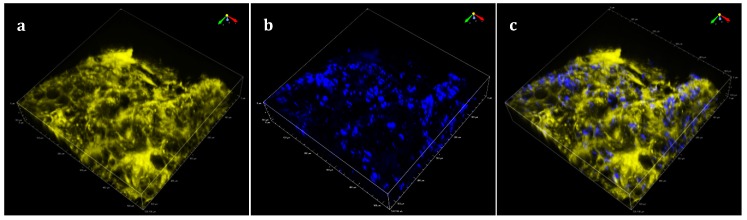
Observation of starch granules embedded in a matrix: (**a**) matrix auto fluorescence signal (yellow); (**b**) SHG signal (blue); (**c**) superposition of SHG and auto fluorescence matrix signals (530 µm × 530 µm × 112 µm). See Table 2 for acquisition parameters.

**Figure 3:**
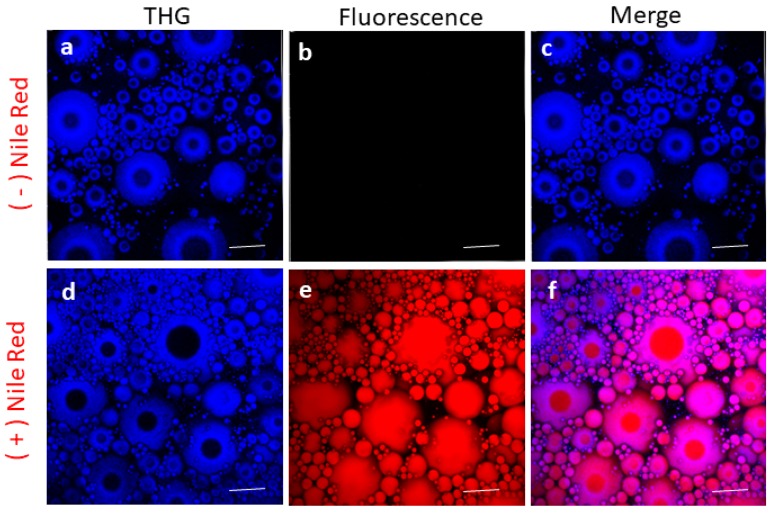
Observation of emulsion lipid droplets: (**a**) third harmonic generation (THG) signal (blue) from emulsion without previous staining; (**b**) endogenous fluorescence; (**c**) merge of (**a**) and (**b**); (**d**) THG signal (blue) from emulsion with previous staining with Nile red; (**e**) red fluorescence from oil stained by Nile red; (**f**) superposition of THG and fluorescence signals. Scale bar 25 µm. See Table 2 for acquisition parameters.

For all images presented, we used color assignments as close as possible to true colors for all detection channels. For instance, SHG from native starch granule, observed in blue channel (410/15), occurred from non-linear light matter interaction at 820 nm (Figure 1b) and at 1040 nm in green channel (525/50), whereby the combined energy of two photons is converted into one emitted photon with one half of the excitation wavelength and doubled energy (Figure 2). THG from interfaces observed in the blue channel (410/15) occurred from non-linear light−matter interaction at 1240 nm (Figure 3b). The combined energy of three photons is converted into one emitted photon with one third of the excitation wavelength and tripled energy (photons collected in the blue channel 410/15), whereas the auto-fluorescence of the matrix was observed in the yellow channel from excitation at 1040 nm (Table 2).

### 2.3. Image Analysis

Starch granules were quantified from SHG and Safranin detection by image analysis with Fiji software [35]. 2D projection was performed to get Zmax projection. Threshold and analyzed particles were performed on five images with both SHG/Safranin detection on a total of 448 starch granules. Gelatinizated starch was used as negative control for SHG images. For SHG detection, starch granules were considered when more than 15% of blue pixels covered the surface area of the granule. The number of starch granules obtained from fluorescence detection and SHG detection was compared. A fitting curve was drawn to get correlation value between the two tested methods. 

## 3. Results

### 3.1. Starch Imaging from Multiphoton Fluorescence and Harmonic Generation Microscopy

Fluorescence staining of native starch granules was firstly analyzed by multiphoton microscopy from Safranin-O labelling. Safranin-O fluorescence was observed from 1040 nm excitation. The two starch granule populations characteristic of starch were visible with large and small green granules (Figure 1a). SHG from native starch granule was observed in the blue channel from non-linear microscopy at 820 nm (emission filter 410/15, Figure 1b). Signals obtained in the green channel (Safranin labelled starch) and in the blue channel (SHG) were merged in Figure 1c. On this figure, it was possible to observe partial superposition between the fluorescence signal and SHG signal with the SHG signal mainly observed at the periphery of the grain, whatever the size of starch granules (Figure 1b,c). 

This result was concordant with our acquisition mode for SHG detection which consist in using linearly polarized light with epi-SHG detection limited to only one angle of polarization. Analysis performed on images from fluorescence/SHG detection of starch granule demonstrated that the number of starch granules detected from SHG and Safranin was highly correlated (R^2^ = 0.9877) with an error of 10.8% for the SHG starch granule detection compared to Safranin staining method. On the other hand, in Figure 1e–g, we demonstrated that SHG was not detected from gelatinized starch.

Fried starchy product was then observed at 820/1040 nm. Auto fluorescence of the matrix, in this case, the cell wall of a potato, was observed in the yellow channel from 1040 nm excitation (Figure 2a). SHG from starch granules was observed in the blue channel from 820 nm illumination (Figure 2b) on an area of 0.3 mm^2^ and 0.11 mm in depth (see Table 2 for illumination and filter used). SHG and fluorescence signals obtained respectively in blue and yellow channels were merged (Figure 2c). A superposition of these two signals showed that SHG from starch could be used to detect the presence of native starch granules in the fried starchy matrix.

### 3.2. Oil imaging from Fluorescence Staining and Harmonic Signal

Lipid droplets were firstly stained in an emulsion by using Nile red staining. Nile red fluorescence was observed from 1040 nm excitation (Figure 3a). THG was observed from 1240 nm illumination (Figure 3b). THG is generated at the interface of lipid bodies/water whereby the combined energy of three photons is converted into one emitted photon with one third of the excitation wavelength and tripled energy (Illumination at 1240 nm, THG collection at 410/15 nm) (Figure 3b). The superposition of Nile red fluorescence and THG signals was illustrated in Figure 3c with a red fluorescence inside the lipid droplets and blue THG at the periphery. Absence of THG was observed at the top of the oil droplets that were in contact with cover glass. 

**Figure 4:**
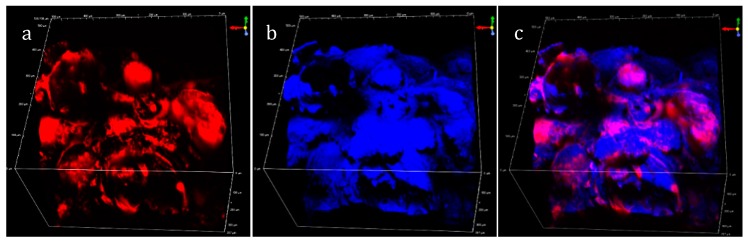
Observation of oil in the fried matrix: (**a**) Nile red fluorescence (red); (**b**) THG signal (blue); (**c**) superposition of THG and fluorescence signals (530 µm × 530 µm × 357 µm). See Table 2 for acquisition parameters.

Imaging of oil in the starchy fried matrix was then performed by using Nile red staining and multiphoton microscopy on an area of 0.3 mm^2^ and 0.36 mm in depth (Figure 4a), The THG signal generated from the sample was illustrated in Figure 4b. The superposition of Nile red fluorescence and THG signals was illustrated in Figure 4c. The superposed areas appeared in purple. THG was not restricted to the lipid area of the product. The blue area corresponding to THG was obtained from the heterogeneities at air/fried product interfaces and was more relevant to give information on the surface topography of the product than oil dispersion in the matrix (Figure 4c).

### 3.3. Distribution of Starch Granules and Surface Topography of Label-Free Fried Starchy Matrix

**Figure 5:**
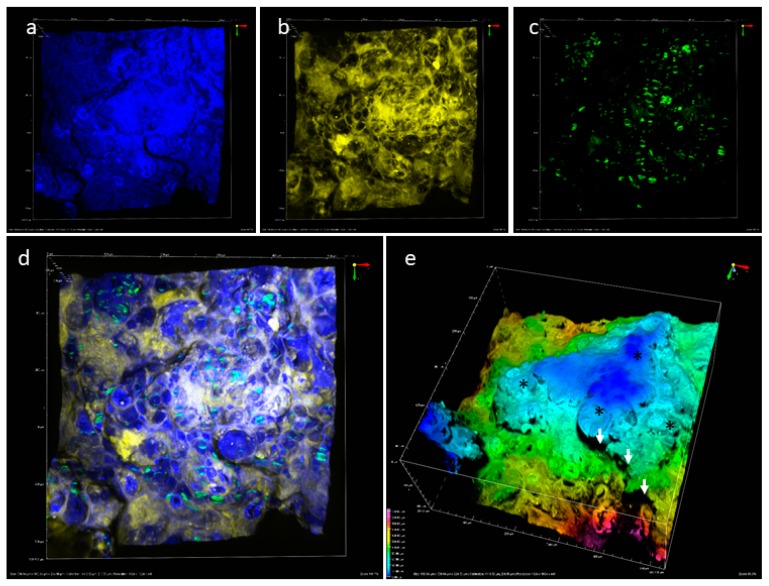
Observation of the matrix: (**a**) THG signal; (**b**) matrix auto-fluorescence signal; (**c**) SHG signal; (**d**) superposition of THG, SHG and matrix auto fluorescence signals; (**e**) 3D imaging of THG topography (530 µm × 530 µm × 224 µm). See Table 2 for acquisition parameters.

The multiphoton analysis of a label-free fried starchy matrix were performed with dual wavelength excitation, respectively 1240 and 1040 nm on an area of 0.3 mm^2^ and 0.22 mm in depth. (Figure 5). THG was observed in the blue channel (laser line 1240 nm), auto-fluorescence in yellow channel (laser line 1040 nm), SHG in the green channel (laser line 1040 nm) (Figure 5a–d).

The resulting 3D image showed an irregular surface of the sample in accordance with the macroscopic aspect of the product. This access to the irregular surface structure of the product was possible thanks to the virtual sectioning in depth facilitated by infrared light. As observed on the oil analysis in the matrix, THG gave information about the surface topography because of the air/matrix interfaces. Therefore, a 3D representation of the THG investigation allowed the visualization protrusions and depressions in the sample (Figure 5e). On topographic image, heterogeneities at the surface of the sample were due to the presence of puffiness and cracks (noted with an arrow and stars). The double exploration of THG and SHG allowed the analysis of the intact starch granule distribution in the first 100 µm of the product and its porosity. (Figure 5d,e).

## 4. Discussion

A label-free starchy fried matrix was observed thanks to multiphoton microscopy with imaging of harmonic generation signals and endogenous auto-fluorescence generated by the components of the sample. Auto-fluorescence of the starchy fried matrix was due to the phenolic compounds of the potato cell contained in dehydrated potato [19,36,37]. SHG from starch granules due to the presence of semi-crystalline polysaccharides have been already described by several authors [24,25,26,38,39]. The SHG-active molecule was amylopectin, which accounts for the crystallinity in starch granule. Cisek et al. [24,25] have previously demonstrated that SHG could be used to follow the change in the hydration of starch, and that a heat treatment resulted in a significant SHG signal decrease. It is in accordance with the low presence of starch granules observed in the fried sample heated at 170 °C and for which the majority of starch granules must have been gelatinized or melted by the high temperature. Bouchon and Pyle [1] suggested that a quick dehydration of native starch granules at the surface of the product could prevent gelatinization. This method we proposed is a new approach to starch analysis in fried starchy matrix that could allow both visualizing straight the impact of different heat treatments or compositions on intact starch granules on the surface and in the depth of the crust [24].

THG obtained thanks to the interface’s heterogeneities of the product gave very interesting data on its surface topography with a 3D approach limited to the surface. Surface structure of the fried food product is usually analyzed by SEM [1,5,40,41], allowing high resolution analysis in 3D with a destructive approach. Non-intrusive analysis of surface structures could be performed by SLM [42] that allows obtaining a topographic map with specific color scale for the z direction representing topographic irregularities. This characterization of the surface topography is an important parameter because it is directly related to the texture and the fragility or solidity of fried product [17,41]. Omidiran et al. [43] also observed that presence of holes in the network could enhance oil migration inside the product. In the fried starchy matrix used to demonstrate the relevance of multiphoton investigation, oil was stained by using Nile red and this staining revealed that oil did not recover the whole surface of the product. This information has to be considered with care because the Nile red solution used for the oil staining was previously prepared with acetone, a solvent that is capable of dissolving oil or displace the fat in the product. It is interesting to note that non-linear microscopy allowed access both to the distribution of intact starch granules and surface topography of food product thanks to SHG and THG respectively. On the other hand, THG was not a tool to image specifically oil in label-free fried starchy matrix. Visualization of puffiness and cracks at the surface of a food product from THG is nevertheless a new interesting approach to analyze microstructure of the final product well known to play a role in the quality attributes.

Finally, from this study, we have demonstrated that SHG and THG could be combined with endogenous fluorescence and fluorescence from specific labeling as oil staining by Nile red fluorescent probe to characterize microstructure of a starchy fried matrix. This approach could provide complementary information on both porosity and its relationship with oil absorption by using oil staining during the frying process which is routinely analyzed to predict the texture of the product [10,42].

## 5. Conclusions

Harmonic generation microscopy shows many advantages compared to other technics, it is a quick and non-destructive method to image intact starch granules after a frying process in the starchy fried matrix and the topography of the surface without any previous sample preparation. This innovative label-free approach could be used to follow the evolution of starch gelatinization process related to the loss of SHG during the transformation of starchy food products and to analyze the surface irregularities of a food product due to THG investigation determinant on food microstructure. SHG detection by using linearly polarized light is limited to about only half of the starch area but we have demonstrated the potentialities of this method from acquisition of Zstack and analysis of maximal projection in 2D. Circularly polarized light would be obviously a best way to investigate entire granule. On the other hand, we demonstrated that THG was not a tool to image specifically oil in label-free fried starchy matrix but could be combine with oil staining for investigation of fat distribution. 

## Figures and Tables

**Table 1:** Stain fluorescence emission wavelengths.

Stain	Emission Wavelength		Ref.
	Minima (nm)	Maxima (nm)	
Safranin O	530	550	[34]
Nile red	638	768	[19]

**Table 2:** Multiphoton settings used in presented work, specifications of filters are provided in brackets with central wavelength and bandwidth.

Constituent	Excitation (nm)	Detection Channel Emission (nm)				Depth (µm)	Number of Images (Step)	Nyquist in Depth
		Blue	Green	Yellow	Red			
**Starch granule** **(Figure 1)**	820	SHG				26.5	53 (0.5 µm)	Yes
	(49.68 mW)	(FF01-415/10-25)						
	1040		Safranin					
	(84 mW)		(FF03-525/50−25)					
**Matrix Starch** **(Figure 2)**	820	SHG				112.5	225 (0.5µm)	Yes
	(86.4 mW)	(FF01-415/10−25)						
	1040			Auto fluo.				
	(135 mW)			(FF03-575/25−25)				
**Oil emulsion** **(Figure 3)**	1240	THG				39	39 (1µm)	No
	(539.5 mW)	(FF01-415/10−25)						
	1040				Nile Red			
	(34.5mW)				(FF01-629/56−25)			
**Matrix Oil** **(Figure 4)**	1240	THG				357	357 (1 µm)	No
	(539.5 mW)	(FF01-415/10−25)						
	1040				Nile Red			
	(30 mW)				(FF01-629/56−25)			
**Matrix: starch, topography** **(Figure 5)**	1240	THG			SHG	224	224 (1 µm)	No
	(380.14 mW)	(FF01-415/10−25)			(FF01-629/56−25)			
	1040		SHG	Auto fluo.				
	(150 mW)		(FF03-525/50−25)	(FF03-575/25−25)				
